# Plasma biomarkers of Alzheimer's disease and related dementias and changes in cognitive function in US and Australian older individuals

**DOI:** 10.1002/alz70856_104041

**Published:** 2025-12-24

**Authors:** Zimu Wu

**Affiliations:** ^1^ Monash University, Melbourne, VIC, Australia

## Abstract

**Background:**

Plasma biomarkers of Alzheimer's disease and related dementias are promising tools for early detection of cognitive impairments. This study examined their associations with changes in cognitive function over time in later life.

**Method:**

Data were obtained from a cohort of community‐dwelling cognitively healthy older individuals aged 65+ years recruited from the US (*n* = 1,181) and Australia (*n* = 11,930). Cognitive function including global cognition, verbal fluency, episodic memory and psychomotor speed was assessed repeatedly for up to 11 years. Plasma biomarkers measured included phosphorylated tau181 (*p*‐tau181), glial fibrillary acidic protein (GFAP), neurofilament light chain (NfL) and amyloid beta 42 and 40 (Aβ42 and 40).

**Result:**

Findings were consistent across the US and Australian participants. Higher plasma levels of *p*‐tau181, GFAP and NfL were associated with a greater decline in global cognitive function, verbal fluency, episodic memory and psychomotor speed (linear β coefficients: ‐0.022 to ‐0.300). However, the association between *p*‐tau181 and verbal fluency was weaker (US: β=‐0.001; Australian: β=‐0.009). In contrast, a larger Aβ42/40 ratio was associated with slower declines in all cognitive outcomes (linear β coefficients: 0.017 to 0.126). Stratified analyses showed consistent trends between subgroups, with slightly stronger associations for *p*‐tau181 observed in females and individuals with chronic kidney disease.

**Conclusion:**

Our findings demonstrate the utility of plasma biomarkers in monitoring cognitive trajectories in cognitively healthy older individuals. Their ability to detect early neurodegeneration and predict cognitive decline has important implications for early intervention and personalized management.

